# Role of Leukocyte Cell-Derived Chemotaxin 2 as a Biomarker in Hepatocellular Carcinoma

**DOI:** 10.1371/journal.pone.0098817

**Published:** 2014-06-03

**Authors:** Hirohisa Okabe, Evan Delgado, Jung Min Lee, Jing Yang, Hiroki Kinoshita, Hiromitsu Hayashi, Allan Tsung, Jaideep Behari, Toru Beppu, Hideo Baba, Satdarshan P. Monga

**Affiliations:** 1 Department of Pathology, University of Pittsburgh, Pittsburgh, Pennsylvania, United States of America; 2 Department of Gastroenterological Surgery, Graduate School of Life Sciences, Kumamoto University, Kumamoto, Japan; 3 Department of Surgery, University of Pittsburgh Medical Center, Pittsburgh, Pennsylvania, United States of America; 4 Department of Medicine, University of Pittsburgh, Pittsburgh, Pennsylvania, United States of America; 5 Department of Multidisciplinary Treatment for Gastroenterological Cancer, Kumamoto University Hospital, Kumamoto, Japan; Institut für Pathologie, Greifswald, Germany, Germany

## Abstract

We sought to identify a secreted biomarker for β-catenin activation commonly seen in hepatocellular carcinoma (HCC). By examination of our previously published genearray of hepatocyte-specific β-catenin knockout (KO) livers, we identified secreted factors whose expression may be β-catenin-dependent. We verified expression and secretion of the leading factor in HCC cells transfected with mutated (Hep3B^S33Y^)-β-catenin. Serum levels of biomarker were next investigated in a mouse model of HCC with β-catenin gene (Ctnnb1) mutations and eventually in HCC patients. Leukocyte cell-derived chemotaxin-2 (LECT2) expression was decreased in KO livers. Hep3B^S33Y^ expressed and secreted more LECT2 in media as compared to Hep3B^WT^. Mice developing HCC with Ctnnb1 mutations showed significantly higher serum LECT2 levels. However patients with *CTNNB1* mutations showed LECT2 levels of 54.28±22.32 ng/mL (Mean ± SD; n = 8) that were insignificantly different from patients with non-neoplastic chronic liver disease (32.8±21.1 ng/mL; n = 15) or healthy volunteers (33.2±7.2 ng/mL; n = 11). Intriguingly, patients without β-catenin mutations showed significantly higher serum LECT2 levels (54.26 ± 22.25 ng/mL; n = 46). While β-catenin activation was evident in a subset of non-mutant β-catenin HCC group with high *LECT2* expression, serum LECT2 was unequivocally similar between β-catenin-active and -normal group. Further analysis showed that LECT2 levels greater than 50 ng/ml diagnosed HCC in patients irrespective of β-catenin mutations with specificity of 96.1% and positive predictive value of 97.0%. Thus, *LECT2* is regulated by β-catenin in HCC in both mice and men, but serum LECT2 reflects β-catenin activity only in mice. Serum LECT2 could be a potential biomarker of HCC in patients.

## Introduction

Primary liver cancer, which is predominantly hepatocellular carcinoma (HCC), is the sixth most common cancer worldwide and the third most frequent cause of cancer mortality [1]. β-Catenin gene (*CTNNB1)* mutations are one of the major oncogenic gene alterations in HCC seen in 10–40%, while mutations affecting Axin1 are seen in around 10% of all HCCs [2]. *CTNNB1* mutations are observed in exon-3 that contain phosphorylation sites essential for β-catenin degradation leading to its stabilization and enhanced expression of target genes such as *glutamine synthetase* (*GS*), *axin2* and *regucalcin* [3], [4], [5], [6]. This mutation is mutually exclusive to p53 mutation, which is the most common mutation in HCC [7], [8]. No routine test is currently available that can yield any genetic information relevant to HCC. Since biopsies for HCC carry high risk because HCCs are usually associated with an underlying liver disease, serum biomarkers specific for a molecular aberration may be highly relevant in personalized medicine in Oncology.

LECT2 is a 16 kDa chemotactic protein purified from human T-cell line [9]. Since hepatocytes are the chief source, expression of LECT2 is specific for liver [10]. LECT2 is a direct target of β-Catenin and has been shown to have a role in the pathogenesis of HCC [11]. In a mouse liver tumor model, LECT2 prohibits tumor progression by regulating Th2-based inflammation [12]. Although its role is well known as chemokine-like secreted protein involved in inflammation, there is no investigation about its serum levels especially in the setting of liver tumor development.

Here, screening a previously published Affymetrix gene array, we identify *Lect2* expression to be decreased in hepatocyte-specific β-catenin knockout livers [13]. Next, using an *in vitro* analysis in human HCC cells, we demonstrate that indeed *LECT2* expression and its protein levels reflect β-catenin activity and hence hypothesize that it may be a good biomarker for HCC with β-catenin activation. The utility of LECT2 as a biomarker was validated first in a mouse liver tumor model where exon-3 mutation in β-catenin gene and ensuing β-catenin activation is implicated in HCC pathogenesis [14], [15]. However, in HCC patients, serum LECT2 levels were not significantly different in tumor with *CTNNB1* or without *CTNNB1* mutations when compared to patients with chronic liver disease or healthy volunteers. Furthermore, despite β-catenin activation observed in an additional subset of non-*CTNNB1* mutated HCC, which showed high *LECT2* expression, serum LECT2 levels were not predictive for active β-catenin signaling in the tumor. Interestingly though, irrespective of molecular aberrations, LECT2 levels were significantly higher in all HCC patients versus patients with cirrhosis or healthy controls. In fact, serum LECT2 ≥ 50 ng/ml indicated HCC with high specificity and positive predictive value.

## Materials and Methods

### Cell lines and treatment

Human HCC cell lines, Hep3B, HepG2, SNU449, SNU398, and HuH7, were obtained from the American Type Culture Collection (Manassas, VA). Cells were cultured in Eagle's minimal essential medium (EMEM) or RPMI supplemented with 10% vol/vol FBS at 37°C in a humidified 5% carbon dioxide atmosphere. For siRNA knockdown experiment, the cells were transfected using Lipofectamine 2000 (Life Technologies, Grand Island, NY) with β-catenin (*CTNNB1*) or scrambled Negative Control siRNAs (Ambion, Grand Island, NY) as previously described [16]. Wild type β-Catenin gene (WT) or β-Catenin gene mutated at serine 33 to tyrosine (S33Y), which is constitutively active, were stably transfected into Hep3B cells to generate Hep3B^WT^ and Hep3B^S33Y^, respectively, as described previously [17].

### Animal studies

All animal experiments were performed under the guidelines of the National Institutes of Health and the Institutional Animal Use and Care Committee at the University of Pittsburgh and approved by the Institutional Animal Use and Care Committee at the University of Pittsburgh. C3H/He mice were injected intraperitoneally with DEN (Sigma-Aldrich, Inc.) at a dose of 90 µg/gram body weight at 6 weeks of age and 3 weeks later putting them on a diet containing 0.05% phenobarbital (PB) until sacrifice at 6–8 months. Serum was collected at time of euthanasia and simultaneously, liver tissues were collected for histology and protein analysis.

### Mutation analysis

Genomic DNA was extracted using DNA Micro Kit (Qiagen, Valencia, CA) from both the frozen liver sections after hematoxilin and eosin (HE) staining (Sigma-Aldrich, Inc.) to identify the tumor and from patient's frozen liver tumor tissues. Amplification of exon 3 of β-catenin gene (*CTNNB1*) using polymerase chain reaction (PCR), gel extraction, purification of PCR products, and direct sequencing were performed as described previously [18].

### Immunohistochemistry and Western blot

Immunohistochemical staining and Western blot was performed as described previously [19]. Rabbit polyclonal anti-GS (Santa Cruz, 1∶100 dilution) was used in immunohistochemistry. Antibodies used in Western blot were mouse monoclonal anti-β-Catenin antibody (Santa Cruz, 1∶1000 dilution), goat polyclonal anti-LECT2 antibody (Santa Cruz, 1∶100 dilution), and rabit polyclonal anti-GAPDH (Santa Cruz, 1∶1000 dilution).

### Real-time polymerase chain reaction

In mice analyses, total RNA was extracted from frozen sections after H&E staining using a mirVana microRNA isolation kit (Ambion) in accordance with the manufacturer's instructions. After reverse transcription and DNase treatment were performed, qRT-PCR was performed on a StepOne Plus using 2× SYBR Green PCR Master Mix (Applied Biosystems, Foster, CA). The primers' sequences are as follows: *Glutamine Synthetase (*encoded by *Glul)* forward, 5′- CTCGCTCTCCTGACCTGTTC -3′ and reverse, 5′- TTCAAGTGGGAACTTGCTGA -3′; LECT2 forward, 5′- CCCACAACAATCCTCATTTCA -3′ and reverse, 5′- GTTAGCCCATGGTCCTGCTA -3′; *GAPDH* was used as an internal control.

In human analyses, total RNA was extracted from frozen tissues and qRT-PCR analysis performed as described previously [20].

### Enzyme-linked immunosorbent assay (ELISA)

Serum LECT2 levels were measured by either human or mouse LECT2 ELISA kit (Medical & Biological Laboratories (MBL) Co, Ltd, Niigata, Japan) according to the manufacture's protocol.

### Clinical tissue and serum samples

All tissues and materials used in this study were obtained under an approved Institutional Review Board protocol at the University of Pittsburgh and Kumamoto University. Specifically, frozen tissues and serum samples were obtained from HCC patients in the Department of Surgery, University of Pittsburgh (Pittsburgh, PA; n = 20) with a written informed consent approved by the University of Pittsburgh Institutional Review Board. Frozen tissues and serum samples from HCC patients were also collected by the Department of Gastroenterological Surgery, Kumamoto University (Kumamoto, Japan; n = 45), with a written informed consent approved by the Institutional Review Board at the Kumamoto University. Additional serum samples were obtained from patients with chronic liver disease (n = 15) in the Department of Medicine that did not have any evidence of HCC as determined by normal serum α-fetoprotein and negative abdominal ultrasound or CT scan within 6 months of serum collection. These patients also signed informed consent prior to providing serum samples and the University of Pittsburgh Institutional review board approved the study.

### Chromatin immunoprecipitation (ChIP)

ChIP Assay was performed using Hep3B^WT^ and Hep3B^S33Y^ cells (Cambridge, MA). 3×10^6^ cells were fixed with 1% formaldehyde for 10 min, incubated with glycine (0.125 M) for 5 min, and then washed twice with PBS. After a short spin, the pellets were resuspended in cell lysis buffer (5 mM PIPES, pH8.0, 85 mM KCl, 0.5% NP-40) by pipetting. After centrifugation, the pellets were resuspended in nuclear lysis buffer (50 mM Tris, pH8.1, 10 mM EDTA, 1% SDS) containing protease inhibitors by pipetting, and sonicated to break chromatin into fragments of around 0.3–1.0 kb length. Subsequent IP experiment was performed with ChIP-IT High sensitivity kit (Active motif, Carlsbad, CA). The diluted DNA-protein complex (25 µg of protein) was incubated overnight at 4°C with different antibodies (rabbit anti-TCF4, Cell Signaling Technology; rabbit IgG and goat anti-HNF1α, Santa Cruz) in the presence of herring sperm DNA and protein A/G agarose beads. PCR was performed using primers for *LECT2* promoter region: 5′- CAGCCCAGAAGACTGTCGAT -3′ (forward) and 5′- GATTAGAGTTGCCCCCACAC -3′ (reverse); albumin promoter region, 5′- TGGAGAAAACAGTTCCAGATGGT -3′ (forward) and 5′- CGTGTGGGGTTGACAGAAGA -3′ (reverse).

### Cell culture and luciferase Assay

SNU449 cells were plated in 24 well plate and treated with either 50 nM control si-RNA or 50 nM β-Catenin si-RNA with lipofectamine iMAX (Invitrogen). TOPFLASH reporter plasmid was transfected using the lipofectamine 2000 (Invitrogen) a day after the siRNA treatment. Subsequently, cells were treated with TGF-β1 (5 ng/mL) (R&D system) for 24 hours. Cells were lysed and prepared using the luciferase reporter assay system (Promega, Madison, WI). Reporter activity was read on a luminometer (Lumat; EG & G Berthold).

### Statistical analysis

Statistical analysis was performed using the JMP 8.0 software (SAS Institute, Cary, NC, USA). Values were presented as the mean ± standard deviation (SD). Differences between two variables were calculated using the Wilcoxon test. Multiple samples were compared by ANOVA followed by Tukey-Kramer post hoc test. P<0.05 was considered to indicate a statistically significant difference.

## Results

### Identification of LECT2 regulation by β-Catenin

**Figure 1:**
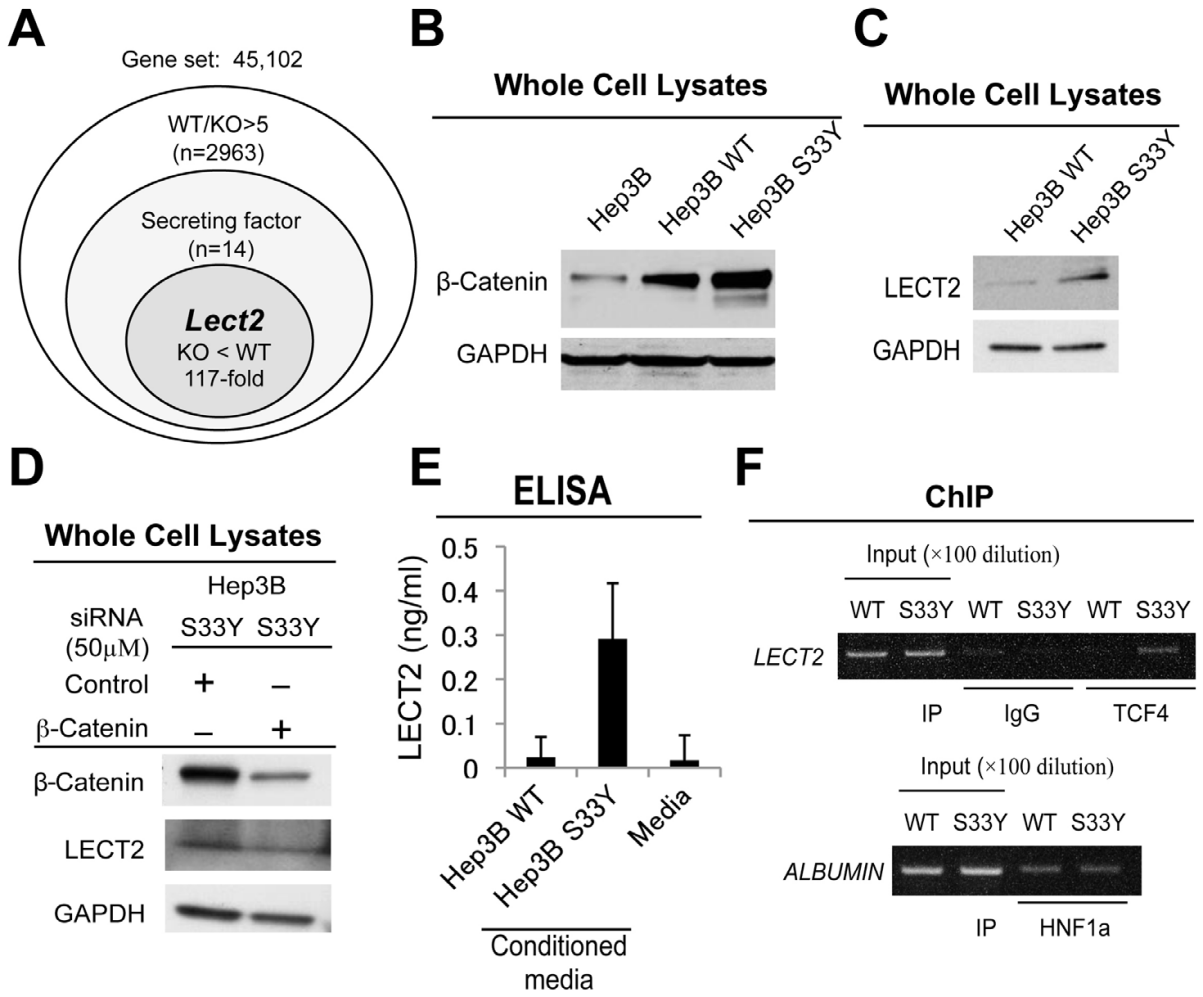
Regulation of LECT2 expression by β-catenin. A. Strategy to identify biomarker for β-catenin activation. Microarray analysis was performed using liver tissue from hepatocyte-specific β-catenin knockout (KO) and wild-type (WT) mice, which identified 14 secreted targets. *Lect*2 expression was 117-fold lower in KO livers. B. β-Catenin expression in Hep3B cells and stable cell lines established with wild-type β-catenin (Hep3B WT)- or mutated β-catenin (Hep3B S33Y)-transfected cells. C. Representative Western blot shows increased LECT2 protein levels in Hep3B S33Y cells as compared to Hep3B WT. D. Hep3B S33Y cells transfected with either *β-catenin* or *control* siRNA showed decreased β-catenin and LECT2 protein levels in a representative Western blot. E. Increased LECT2 protein levels were observed in culture media collected from Hep3B S33Y cells as compared to Hep3B WT as analyzed by ELISA. Basal media was used as a negative control. F. Occupation of *Lect2* promoter by TCF4 especially in Hep3B S33Y cells was as assessed by ChIP. Albumin promoter is not regulated by β-catenin but by HNF1α, which is used as quality control for chromatin.

To identify specific biomarkers for β-catenin upregulation, we utilized our previous dataset of microarray analysis comparing gene expression of liver tissue of hepatocyte-specific β-catenin knockout (KO) with that of wild type (WT) [13]. From a list of 2963 upregulated genes in WT as compared to KO (with fold change > 5), we identified 14 genes that encode for secreted proteins (Table 1). The leading candidate *lect2* showed a 117-fold lower expression in KO as compared to WT (Figure 1A). To validate if *LECT2* expression could be induced by β-catenin activation, we used two previously generated stable human cell lines, Hep3B cells expressing wild type β-catenin (Hep3B^WT^) and Hep3B cells expressing S33Y-mutated β-catenin (Hep3B^S33Y^), the latter showing highest β-catenin levels (Figure 1B) [17]. We compared *LECT2* expression between these cell lines using qRT-PCR analysis and detected a notable increase in its expression in Hep3B^S33Y^ cells (data not shown). Whole cell lysates also showed increased LECT2 protein levels in Hep3B^S33Y^ as compared to Hep3B^WT^ cells (Figure 1C). To see if LECT2 upregulation is caused by active β-catenin, Hep3B^S33Y^ were transiently transfected with β-catenin or control siRNA. β-Catenin knockdown led to a notable decrease in LECT2 protein levels (Figure 1D).

**Table 1:** Candidate genes encoding secreted protein.

Gene	Name
*Lect2*	leukocyte cell-derived chemotaxin 2
*AFP*	alpha fetoprotein
*Slpi*	secretory leukocyte protease inhibitor
*Del1*	del1 minor splice variant
*Clecsf8*	C-type lectin, superfamily member 8
*Sh2d1a*	SH2 domain protein 1A
*Wnt10a*	wingless related MMTV integration site 10a
*IL22b*	interleukin-22b
*Egf*	epidermal growth factor
*Fgf13*	fibroblast growth factor-related protein FGF-13
*Wnt4*	wingless-related MMTV integration site 4
*Igf2*	insulin-like growth factor 2
*Fdp*	fibrocyte-derived derived protein
*Scya24*	eotaxin-2

Next, to assess if LECT2 is being secreted, conditioned media was collected from Hep3B^WT^ and Hep3B^S33Y^ cells and subjected to ELISA assay. Significantly higher LECT2 protein was detected in conditioned media from Hep3B^S33Y^ (0.29±0.13 ng/mL) as compared to Hep3B^WT^ cells (0.023±0.046 ng/mL) (Figure 1E). Basal media, used a negative control, also showed lack of any LECT2 protein (0.017±0.057 ng/mL).

Lastly, to specifically determine if β-catenin-T cell factor (TCF) signaling was indeed regulating *LECT2* expression, chromatin immunoprecipitation (ChIP) analysis was performed on extracts from Hep3B^S33Y^ and Hep3B^WT^ cells. As shown in figure 1F, *LECT2* was pulled down with TCF4 and not control IgG in Hep3B^S33Y^ cells. This indicates promoter activity driven by TCF4-β-catenin in Hep3B^S33Y^ cells. *Albumin*, whose expression is β-catenin-TCF independent, was pulled down by HNF1α in both Hep3B^S33Y^ and Hep3B^WT^ cells. These results show occupancy of *Lect2* promoter by TCF4, thus demonstrating its regulation by the β-catenin-TCF signaling.

### HCC with β-Catenin mutation increases serum LECT2 level in mice

**Figure 2:**
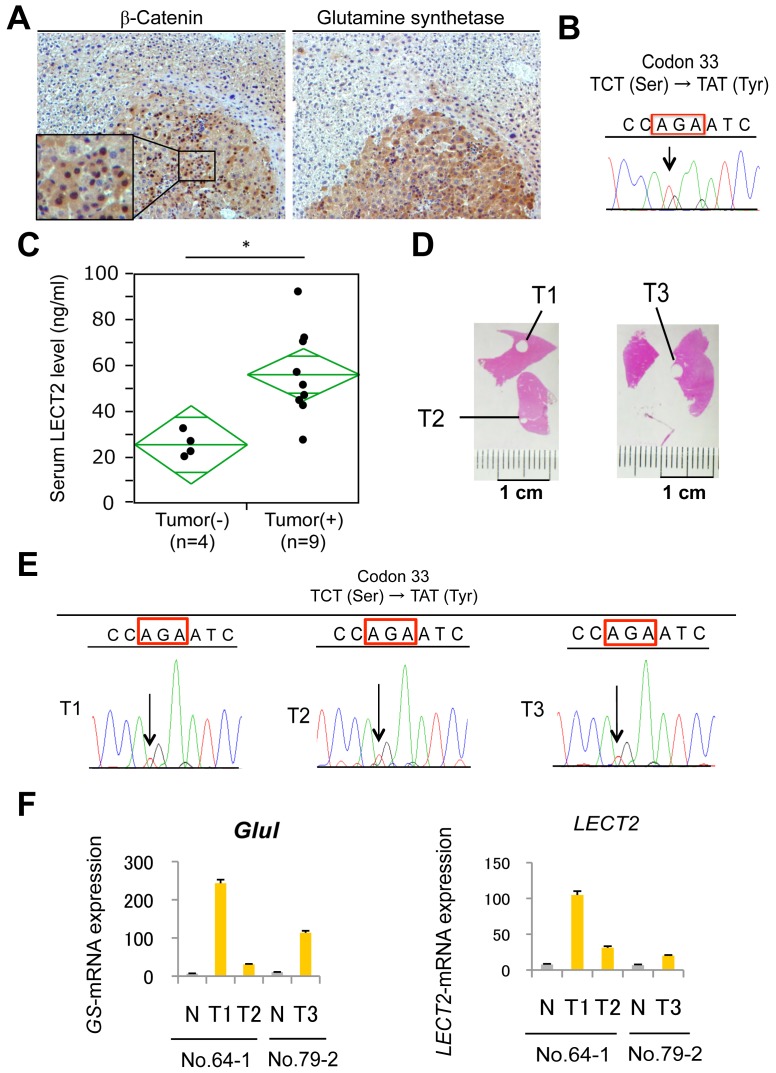
Serum LECT2 levels in mice with β-catenin gene mutated HCC. A. Representative picture of β-catenin (left panel) and GS (right panel) immunohistochemistry of liver of a tumor bearing mouse at 8 months after DEN/PB treatment. Magnification, 100×. B. Using frozen tissue from a representative tumor, β-catenin gene exon-3 mutation affecting codon 33 (red box) was confirmed by direct sequencing. C. Serum LECT2 levels were significantly (*) increased in tumor bearing versus non-tumor bearing DEN/PB treated mice as analyzed by ELISA. (* *p*<0.01). D. Representative pictures of frozen sections from which tumors (T1-T3) were scraped for direct sequencing. E. Sequence analysis from three tumor lesions (T1-T3) show S33Y-β-catenin gene mutations in codon 33 (red boxes) by direct sequencing. F. *Glutamine Synthetase (Glul)* and *Lect2* expression in three tumor lesions (T1-T3) were assessed by qRT-PCR. Gene expression of background liver tissues surrounding tumor are shown as N.

To investigate if Lect2 could be a serum biomarker in mice, we used a murine HCC model, which utilizes β-catenin signaling as a major mechanism of carcinogenesis. Tumor induction by a single injection of diethylnitrosamine followed by exposure to PB as described in methods and elsewhere has been shown to select for exon-3 mutations in β-catenin gene to give rise to HCC at 6-8 months [14], [15]. Indeed, HCC observed in mice with this protocol were strongly GS-positive and had nuclear β-catenin accumulation as observed by immunohistochemical staining (Figure 2A). Genetic alteration in β-catenin gene contributing to β-catenin activation was confirmed by direct sequencing (Figure 2B). We could recognize liver tumor formation in 9 mice out of 13 that were subjected to DEN/PB protocol. Strikingly, the 9 mice with evidence of histological tumor burden showed significantly (*p*<0.01) higher serum Lect2 levels (55.9±19.9 ng/mL) as compared to the 4 non-tumor bearing mice (24.9±5.5 ng/mL) (Figure 2C). To confirm mutations in β-catenin gene due to existing tumor heterogeneity and also verify corresponding *Lect2* expression, we extracted both genomic DNA and total RNA from same nodules (Figure 2D). Direct sequencing showed a common mutation (S33Y) in T1, T2 and T3 nodules (Figure 2E). We next examined *Lect2* mRNA expression as well as *Glul* mRNA (encoding GS protein) expression in these tumor nodules. All 3-tumor nodules had high *Lect2* and *Glul* expressions as compared to the background liver (Figure 2F). Thus, in mice *Lect2* expression and eventually its secretion is upregulated by β-catenin mutations in HCC, which can be detected in serum and hence may be a useful biomarker in this mouse model.

### Serum LECT2 level is inconsistent with β-Catenin mutations in human HCC

**Figure 3:**
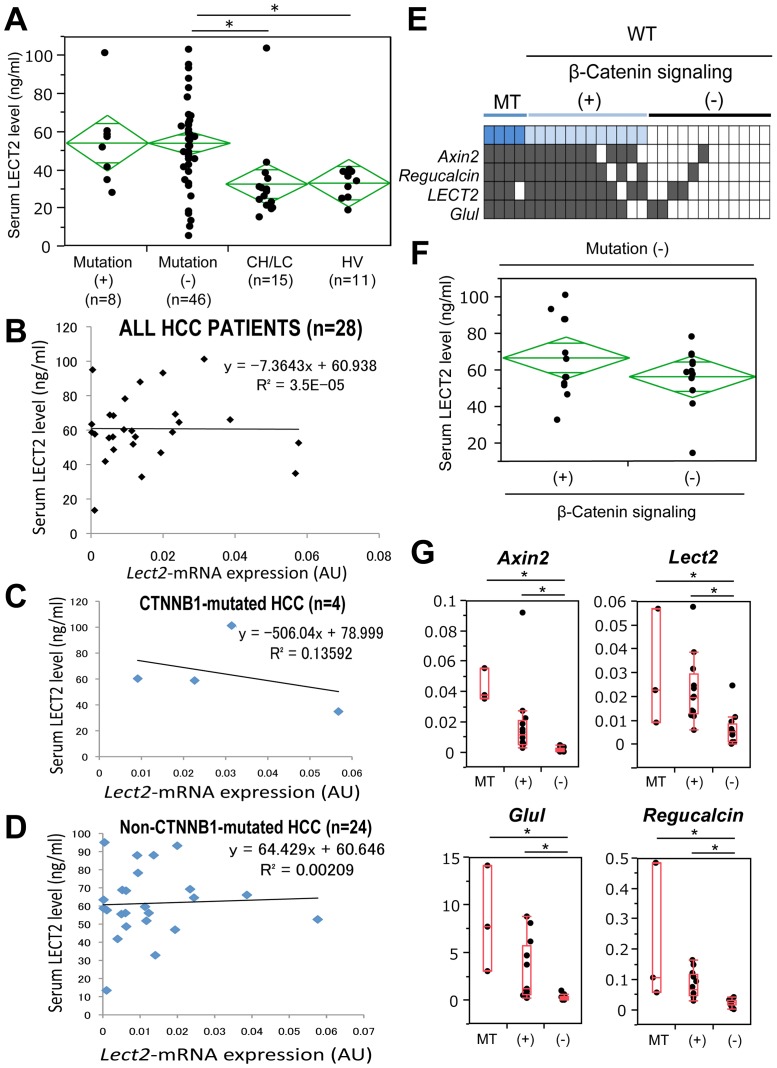
No correlation of β-catenin mutations or β-catenin activation to serum LECT2 levels in patients. A. Serum LECT2 levels in patients with HCC with *CTNNB1* mutations, absent *CTNNB1* mutations, patients with chronic liver fibrosis (CH/LC), and healthy volunteer (HV) as assessed by ELISA. (*p<0.05). B. No correlation observed between *LECT2* expression in tumor and serum levels of LECT2 in HCC patients (n = 28). C. No correlation observed between *LECT2* expression in β-catenin mutated tumors and serum levels of LECT2 in these HCC patients (n = 4). D. No correlation observed between *LECT2* expression in non-β-catenin mutated tumors and serum levels of LECT2 in these HCC patients (n = 24). E. Heat map shows expression of β-catenin target genes in β-catenin-mutated (MT) and non-mutated wild-type (WT) HCC patients (n = 28). Genes assessed included *AXIN2*, *REGUCALCIN*, *LECT2*, and *GLUL*. (+) indicates β-catenin activity as seen by increased expression of at least 2 target genes whereas (−) indicates absent β-catenin activation reflected by lack of target gene expression. F. Serum LECT2 levels showed insignificant difference in HCC patients that lacked β-catenin gene mutations but showed high expression of β-catenin target genes versus patients who have neither β-catenin gene mutations nor any increase in β-catenin target gene expression. G. β-Catenin target gene expression shown by qRT-PCR. Steel-Dwass test was performed to compare the values among three groups. *, *p*<0.05. (+) indicates β-catenin activity as seen by increased expression of at least 2 target genes whereas (−) indicates lack of β-catenin activity due lack of target gene expression.

Sera were available from 54 HCC patients through appropriate IRB approvals. Eight of the 54 patients showed β-catenin gene (*CTNNB1)* alterations in the form of missense mutations in exon-3 (Table 2). Remaining 46 patients lacked any genetic alterations in exon-3 of *CTNNB1.* Additionally, we enrolled healthy volunteers and also patients with cirrhosis due to chronic liver disease (Table 3) to determine serum LECT2 levels and address its efficacy as a tumor marker. Based on Tukey-Kramer post hoc test, serum LECT2 levels in patients with mutated β-Catenin (54.28±22.32 ng/mL; n = 8) were not statistically different from either patients with cirrhosis (32.8±21.1 ng/mL, *p* = 0.091; n = 15) or healthy volunteers (33.2±7.2 ng/mL, *p* = 0.137; n = 11). On the other hand, patients who did not harbor *CTNNB1* mutations showed significantly higher LECT2 level (54.26±22.25 ng/mL; n = 46) than those with cirrhosis and from healthy volunteers (*p* = 0.0044 and 0.0176, respectively) (Figure 3A).

**Table 2:** Clinical characteristics of patients with HCC.

Patient	Age	Gender	Tumor size (cm)	Differentiation status	Serum LECT2 level (ng/mL)	Mutation	RNA available
K284	81	M	4.1	Well	93.2	WT	○
K285	64	M	1.7	Well	87.9	WT	×
K286	69	F	4.9	Moderate	48.7	WT	○
K287	62	M	1.6	Well	62.6	WT	○
K288	63	M	1.7	Well	52.6	WT	○
K289	58	M	3.8	Moderate	83.3	WT	×
K290	77	M	1.5	Moderate	64.6	WT	○
K291	52	M	7.8	Moderate	63.4	WT	○
K293	39	M	2.0	Moderate	69.3	WT	○
K294	36	M	2.5	Moderate	32.8	WT	×
K295	74	M	4.0	Moderate	78.2	WT	○
K296	76	M	2.4	Moderate	46.9	WT	○
K297	70	M	4.4	Necrosis (TACE)	95.1	WT	×
K298	66	M	4.3	Moderate	54.4	WT	×
K299	67	M	2.5	Moderate	61.3	WT	○
K300	74	F	5.5	Moderate	32.8	WT	×
**K301**	**64**	**M**	**3.5**	**Moderate**	**57.2**	**MT**	**×**
**K302**	**86**	**M**	**5.5**	**Poor**	**51.8**	**MT**	**○**
K303	65	M	2.3	Moderate	41.8	WT	○
K304	76	F	1.0	Well	56.1	WT	○
**K305**	**77**	**F**	**2.5**	**Mod**	**34.9**	**MT**	**○**
K306	73	M	1.0	Well	56.1	WT	○
K307	72	M	2.3	Moderate	64.2	WT	×
K308	66	M	11.0	Moderate	66.1	WT	○
K309	72	M	6.8	Moderate	58.7	WT	○
**K310**	**76**	**M**	**4.6**	**Well**	**101.3**	**MT**	**○**
K311	68	F	9.0	Moderate	45.6	WT	×
K312	60	M	1.3	Poor	49.3	WT	×
K313	82	M	4.0	Moderate	34.7	WT	×
**K314**	**83**	**F**	**4.5**	**Moderate**	**59.5**	**MT**	**×**
K315	62	M	4.5	Moderate	57.7	WT	○
K316	75	M	4.0	Moderate	68.4	WT	○
K317	82	F	7.0	Poor	56.0	WT	○
**K318**	**69**	**M**	**3.0**	**Moderate**	**58.8**	**MT**	**○**
K319	63	M	4.0	Moderate	55.5	WT	○
**K320**	**67**	**M**	**2.2**	**Well**	**60.3**	**MT**	**○**
K321	59	M	2.2	Moderate	88.0	WT	○
K322	63	M	6.0	Moderate	13.4	WT	○
K323	69	M	3.3	Moderate	68.8	WT	○
K324	64	M	3.9	Moderate	56.7	WT	○
K325	66	M	3.0	Moderate	61.3	WT	×
K326	77	M	2.5	Poor	47.8	WT	×
K327	70	M	7.6	Moderate	31.9	WT	×
K328	56	M	6.1	Moderate	103.3	WT	×
K329	76	M	4.0	Moderate	56.1	WT	×
P318	58	M	10.6	Moderate	17.2	WT	×
P336	76	F	2.5	Moderate	10.2	WT	×
P328	57	F	4.2	Moderate	5.5	WT	×
P513	72	M	-	Moderate	38.9	WT	×
P520	69	M	5.0	Moderate	18.5	WT	×
P788	56	M	14.0	Moderate	42.7	WT	×
**P958**	**71**	**M**	**17.0**	**Moderate**	**28.4**	**MT**	**×**
P1335	64	M	2.5	Well	49.8	WT	×
P1342	68	M	2.2	Moderate	26.2	WT	×

**Table 3:** Clinical characteristics of control patients with cirrhosis but no HCC.

Patient	Age	Gender	Etiology of Cirrhosis†	T-Bil (mg/dL)	PT-INR	Albumin (mg/dL)	Platelet (×10^4^/mL)
LV0770	45	M	HCV/ETOH	0.3	1.0	4.5	23.6
LV0771	58	F	NASH	0.5	1.2	3.4	13.9
LV0736	54	F	ETOH	0.5	1.1	4.3	20.2
LV0735	62	F	NASH	0.6	1.1	4.3	23.8
LV0697	54	F	ETOH	0.7	1.2	4.5	24.8
LV0744	50	F	HCV	0.8	1.3	4.0	4.4
LV0773	46	M	ETOH	0.8	1.3	3.6	7.7
LV0721	55	F	HCV/ETOH	1.1	1.2	3.8	3.9
LV0708*	64	M	ETOH	1.3	1.3	3.2	9.3
LV0740	55	F	ETOH	1.6	1.0	4.4	16.2
LV0687	58	F	HCV	1.8	1.3	3.0	6.9
LV0711	55	F	ETOH	1.8	1.6	3.5	21.5
LV0714	35	M	Unknown	2.3	1.4	3.4	7.1
LV0710	47	M	ETOH	2.7	1.7	3.5	5.9
LV0703	41	M	AIH	4.0	1.7	3.5	2.8

† HCV, hepatitis type C virus; ETOH, alcoholic; NASH, non-alcoholic steatohepatitis; AIH, autoimmune hepatitis.

*serum LECT2 level value was very high (103.9 ng/mL).

To address if serum LECT2 levels showed any correlation with *LECT2* expression in the tumors, we selected a group of 28 patients for which had serum and corresponding tumor tissue (Table 2). Intriguingly, no correlation of serum LECT2 to its mRNA expression was detectable (Figure 3B). Even upon stratification of tumors for presence (n = 4) or absence (n = 24) of β-catenin gene mutations, no correlation was evident between serum LECT2 and its gene expression (Figure 3C–D).

This led us to further investigate if a subset of these tumors with WT β-catenin gene may still have β-catenin activation. We examined expression of several surrogate Wnt target genes such as *AXIN2*, *REGUCALCIN*, *LECT2* and *GLUL* in all 28 tumors using qRT-PCR. Based on the known heterogeneity in Wnt signaling in HCC, we labeled a tumor to be β-catenin-active if at least 2 of 4 target genes were simultaneously upregulated. In addition to the 4 tumors with *CTNNB1* mutations, 12 more samples showed increased expression of target genes indicative of β-catenin activation as shown in a heat map (Figure 3E). Based on this classification, we next compared serum LECT2 levels between patients with WT β-catenin gene with active Wnt signaling (+) and with WT β-catenin gene with absent Wnt signaling (−). While some HCC patients in the former group showed high serum LECT2 levels, there was no statistical difference between the two groups (Figure 3F).

Lastly, we compared β-catenin target gene expression among the three HCC groups; 1) patients with *CTNNB1* mutations (MT), 2) patients with WT β-catenin gene but active Wnt signaling (+), and 3) those with WT β-catenin gene but lacking Wnt signaling (−). Patients in MT and (+) groups were insignificantly different from each other in expression values of *AXIN2, REGUCALCIN, LECT2* and *GLUL*, although the expression was always highest in the MT group (Figure 3G). However, both these groups showed significantly higher expression of these four target genes when compared to the (−) group clearly demonstrating lack of active Wnt signaling in this subgroup of HCCs (Figure 3D). Intriguingly, (−) group also showed significantly lower *LECT2* expression than MT and (+) (Figure 3G).

Taken together, these data indicate that despite some HCC patients showing low *LECT2* expression in tumors, they unexpectedly still showed high serum LECT2 level with the values ranging from 50–80 ng/mL.

### Increased serum LECT2 is a biomarker of HCC-independent of β-catenin mutation/activation in tumors

**Figure 4:**
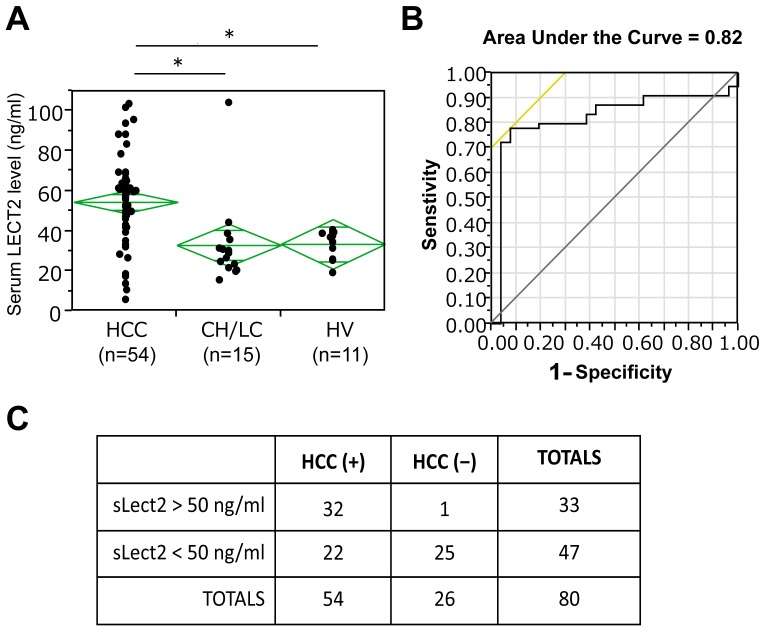
The role of serum LECT2 level as a diagnostic biomarker in HCC. A. Serum LECT2 levels in all HCC patients as compared to patients with chronic liver fibrosis (CH/LC), and healthy volunteer (HV) as assessed by ELISA. (*p<0.01). B. ROC analysis for the utility of LECT2 as a diagnostic marker of HCC with AUC = 0.82. C. Fisher's Exact test shows that based on the cut-off value of serum LECT2 level at 50 ng/mL, sensitivity, specificity, positive predictive value, negative predictive value for the diagnosis of HCC were 59.3%, 96.1%, 97.0%, and 53.2%, respectively.

Next, due to heterogeneity in HCC, we investigated if serum LECT2 levels may be a biomarker of HCC irrespective of a precise molecular basis. Intriguingly, Tukey-Kramer analysis revealed a significant difference in serum LECT2 levels in HCC patients when compared to chronic liver disease patients with cirrhosis (p = 0.0017) and healthy volunteers (p = 0.0078) (Figure 4A). Area Under the Curve (AUC) of 0.82 also highlights its value as a general biomarker of HCC (Figure 4B). A sub analysis was done next with LECT2 serum value cut-off at 50 ng/ml. Fisher Exact test showed significant utility of using >50 ng/ml of serum LECT2 as an indicator of HCC (two tailed p value< 0.0001) (Figure 4C). Serum LECT2 levels of greater than 50 ng/ml were able to detect HCC with a sensitivity of 59.3%, specificity of 96.1%, positive predictive value of 97.0% and negative predictive value of 53.2%.

## Discussion

LECT2 is a secreted protein from hepatocytes functioning like a chemokine, and is known to be directly regulated by β-catenin [11], [12]. We validated the regulation of this important immune regulator by the Wnt signaling in HCC. Using hepatocyte-specific β-catenin KO livers as well as Hep3B cells expressing stable form of β-catenin, we show that *LECT2* expression was indeed β-catenin-dependent. The major goal of the study was to investigate if serum LECT2 levels could reflect β-catenin activity in HCC and hence could be a biomarker to detect HCC-driven by Wnt signaling for prognostic and therapeutic implications. β-Catenin therapeutic targeting is indeed actively discussed in HCC [21], [22]. At the same time β-catenin inhibition in all HCCs may be counterproductive due to a paradoxical observation of enhanced liver injury, inflammation, fibrosis and HCC made in KO exposed to chemical carcinogen, thus making careful selection of patients of high relevance [23], [24]. Since liver-biopsies may not be feasible in many HCC patients due to cirrhosis that usually co-exists, immunohiostochemistry for assessment of β-catenin and/or GS localization as an indicator of *CTNNB1* mutations is often not possible. Thus there is a significant need for biomarker discovery for personalized medicine to identify a subset of HCC patients with β-catenin activation. We hypothesized that serum LECT2 may be a non-invasive way to identify β-catenin gene mutation-harboring HCC. Indeed previous investigations have shown LECT2 to be observed systemically, suggesting that it can be a serum predictor [25], [26].

Using mouse model, we confirmed that serum LECT2 levels indeed correlated with upregulation of *Lect2* expression in the tumor that occurred secondary to β-catenin gene mutations in a murine HCC model [14]. Based on these observations, we propose that serum Lect2 levels in mice may be an excellent non-invasive and simple modality to monitor tumor burden due to β-catenin gene mutations. Since anti-β-catenin drug discovery is a timely concept, and models like DEN/PB may be of essence in testing anti-β-catenin therapies, our studies demonstrate the suitability of assessing serum Lect2 levels in such models to monitor tumor response to experimental therapies.

In patient analysis however, serum LECT2 levels were not statistically different in *CTNNB1*-mutated versus non-mutated group. In fact, we did not find any correlation between expression of *Lect2* in the tumors and its corresponding serum levels in a patient. Since, β-catenin activation in HCC can be due to multiple independent mechanisms such as overexpression of Wnt inhibitors, increased expression of Wnt3 and Frizzled-7, TGF-β activation or other reasons [2], [7], [8], [17], [22], [27], [28], we also examined if serum LECT2 may in fact reflect β-catenin activity rather than its mutational status. Based on gene expression analysis, we also identified patients that showed upregulation of β-catenin target genes but did not harbor any *CTNNB1* mutations in exon-3. While, we did observe β-catenin activation in around 50% more HCC samples, LECT2 serum levels remained ambiguous in this subset as well.

Based on the observation of relatively high levels of LECT2 in serum of a major subset of HCC patients, irrespective of molecular aberration, we were able to address its utility as a general biomarker for HCC. A recent study also identified an increase in serum LECT2 levels in obese individuals and patients with fatty liver [29]. Since we had only two cirrhosis patients with NASH and without HCC in our control group, we are unable to study such correlation in our dataset. Also, we did not attempt to address any biological implications of Lect2 in HCC in the current study. However, Lect2 was recently shown to regulate Th2-based inflammation in HCC [12]. In our analysis, we were unable to detect any correlation between serum LECT2 levels and differentiation status of the tumor.

Thus, in summary we have identified *Lect2* upregulation in HCC, both downstream of *CTNNB1* mutations and due to mutation-independent β-catenin activation. While LECT2 serum levels coincide with *Ctnnb1* mutations in mice, this relationship was ambiguous in patients and *Lect2* expression and serum levels did not correlate with β-catenin activation due to either mutations or otherwise. However, serum LECT2 by itself turned out to be an important biomarker of HCC (AUC  = 0.82). Further, serum LECT2 levels of greater than 50 ng/ml successfully diagnosed HCC in patients irrespective with specificity of 96.1% and positive predictive value of 97.0% and thus should be investigated prospectively for its diagnostic value.
